# Myxofibrosarcoma harboring an *MLH1* pathogenic germline variant associated with Muir-Torre syndrome: a case report

**DOI:** 10.1186/s13053-021-00192-z

**Published:** 2021-08-21

**Authors:** Makoto Nakagawa, Eisuke Kobayashi, Masayoshi Yamada, Tomoko Watanabe, Makoto Hirata, Noriko Tanabe, Mineko Ushiama, Hiromi Sakamoto, Chiaki Sato, Taisuke Mori, Akihiko Yoshida, Teruhiko Yoshida, Kokichi Sugano, Akira Kawai

**Affiliations:** 1 Department of Musculoskeletal Oncology and Rehabilitation, National Cancer Center Hospital, 5-1-1 Tsukiji, Chuo-ku, 104-0045 Tokyo, Japan; 2 Department of Orthopaedic Surgery, Graduate School of Medical Sciences, Kyushu University, Fukuoka, Japan; 3 Endoscopy Division, National Cancer Center Hospital, Tokyo, Japan; 4 Department of Genetic Medicine and Services, National Cancer Center Hospital, Tokyo, Japan; 5 Department of Clinical Genomics, National Cancer Center Research Institute, Tokyo, Japan; 6 Department of Diagnostic Pathology, National Cancer Center Hospital, Tokyo, Japan; 7 Oncogene Research Unit/ Cancer Prevention Unit, Tochigi Cancer Center Research Institute, Tochigi, Japan

**Keywords:** Myxofibrosarcoma, Muir-Torre syndrome, Lynch syndrome, Mismatch repair (MMR), Microsatellite instability (MSI)

## Abstract

**Background:**

Muir–Torre syndrome (MTS), which accounts for a small subset (1–3 %) of Lynch syndrome (LS), is an autosomal dominant genetic disorder characterized by sebaceous gland or keratoacanthoma associated with visceral malignancies. Most families with MTS have pathogenic germline variants (PGV) in *MSH2*. Sarcomas are not common on the LS tumor spectrum, and sarcomas associated with MTS are extremely rare.

**Case presentation:**

Here we report a myxofibrosarcoma of the abdominal wall in a 73-year-old man with a sebaceoma that occurred synchronically, leading to a diagnosis of MTS. The loss of MLH1 and PMS2 protein expression was detected in immunohistochemistry, and high-frequency microsatellite instability (MSI-H) was also confirmed. A germline genetic analysis revealed that he harbored the *MLH1* PGV.

**Conclusions:**

This is the first case of MSI-H myxofibrosarcoma with MTS in an *MLH1* PGV carrier. Although rare, we should recognize that sarcomas can be part of the spectrum of LS and MTS.

**Supplementary Information:**

The online version contains supplementary material available at 10.1186/s13053-021-00192-z.

## Background

Muir–Torre syndrome (MTS), which accounts for a small subset (1–3 %) of hereditary non-polyposis colorectal cancer (HNPCC), also known as Lynch syndrome (LS), is an autosomal dominant genetic disorder characterized by sebaceous gland or keratoacanthoma associated with internal malignancies and often occurs before 50 years of age [1, 2].

The characteristic sebaceous tumors of MTS include sebaceous adenoma, sebaceoma, and less commonly, sebaceous carcinoma [1]. These LS- or MTS-associated tumors often show microsatellite instability (MSI) caused by pathogenic germline variants (PGV) in one of four DNA mismatch repair (MMR) genes—*MLH1*, *MSH2*, *MSH6*, and *PMS2*—resulting in increase in errors during DNA replication and tumorigenesis. In contrast to LS, in which *MLH1* and *MSH2* are equally involved as causative genes, MTS is primarily characterized by a PGV in *MSH2* [3, 4]. Internal malignancies associated with LS and MTS include colorectal, endometrial, ovarian, small intestinal, and genitourinary malignancies [1]. Sarcomas are not considered part of the common LS tumor spectrum, and only occasional LS-associated sarcomas have been reported [5–9]. Furthermore, soft tissue sarcomas associated with MTS are extremely uncommon, and myxofibrosarcoma associated with MTS has not been previously reported [5, 9–11].

Myxofibrosarcoma is one of the most common sarcomas arising in the extremities of elderly patients, with a slight predominance in males [12]. This tumor comprises a spectrum of malignant fibroblastic neoplasms with variably myxoid stroma, pleomorphism, and a distinctive curvilinear vascular pattern [12]. Local recurrence occurs in 30–40 % of cases, usually as a result of inadequate surgery [12, 13]. Infiltrative growth is characteristic in myxofibrosarcoma, correlating with superficial tumors and a positive surgical margin and resulting in worse local control and a poor metastasis-free survival rate [14].

We herein report a myxofibrosarcoma of the abdominal wall in a 73-year-old man with a sebaceoma occurred synchronic, leading to a diagnosis of MTS. This is the first report of myxofibrosarcoma associated with MTS harboring the *MLH1* germline variant.

## Case presentation

**Fig. 1:**
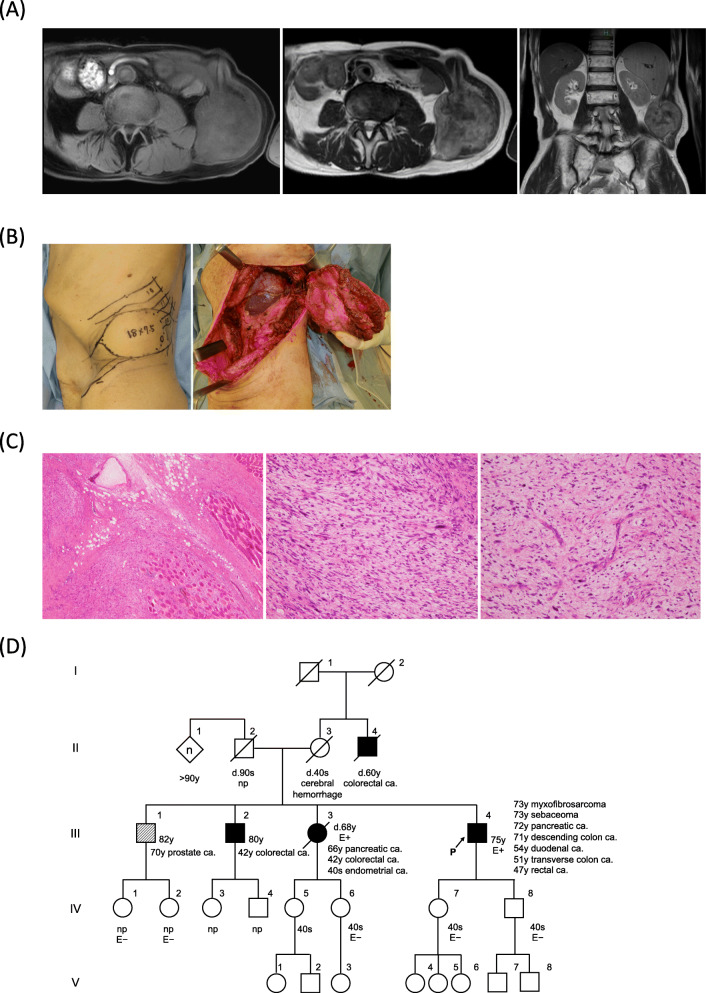
Myxofibrosarcoma arising in the retroperitoneum of a 73-year-old man. **A** Representative magnetic resonance imaging findings of the tumor left abdominal wall. (Left) axial T1-, (middle) axial T2-, and (right) coronal T2-weighted images. **B** Gross images during surgery. (left) Preoperative skin incision design on the left abdominal wall. (right) After wide resection. Note that the 10th to 12th ribs, diaphragm, and peritoneum were resected in combination with the tumor. The spleen was preserved because the tumor had not invaded it grossly. **C** Hematoxylin eosin staining of the excised tumor. (left) Representative histology of the tumor, located mainly in the muscles and extended to the adipose tissues. (middle) Pleomorphic and spindled tumor region in a myxoid stroma. (right) Tumor region focally showing curvilinear vessels with a prominent myxoid matrix. **D** Family pedigree. Squares denote male family members, circles denote female family members, solid symbols show individuals affected by cancer, the arrow denotes the proband, a symbol with a slash indicates a deceased person (with the age at death), and the types of primary tumors are listed beside the symbols. P: proband, E: evaluation (*MLH1* c.1233_1254dup22)

A 73-year-old Japanese man presented with a 2-month history of a mass at the left abdominal wall. Magnetic resonance imaging (MRI) demonstrated a 7.5 × 7.4 × 6.6-cm tumor involving the left 10th and 11th ribs and extending to the surface of the paraspinal muscles and quadratus lumborum. The tumor showed iso-intensity with muscles on T1-weighted imaging and mixed high and low intensity on T2-weighted imaging (Fig. 1A). He subsequently underwent positron emission tomography/computed tomography (PET-CT), and the standardized uptake value (SUV_max_) of the tumor was 3.96. No regional or distant metastases were identified.

As a needle biopsy confirmed a diagnosis of sarcoma, wide resection and abdominal wall reconstruction with a pedicled anterolateral thigh flap were performed (Fig. 1B). Histologically, pleomorphic and spindled tumor cells with often atypical mitoses proliferated in a myxoid stroma (Fig. 1C). The tumor also focally showed elongated capillaries with a prominent myxoid matrix. Necrosis was also observed. On immunohistochemistry, desmin, S100, αSMA, myogenin, and MDM2 were negative. The final diagnosis was high-grade myxofibrosarcoma.

Four months after the initial operation, the patient noticed a 7.0-mm cutaneous tumor on his left upper arm. The tumor was resected and diagnosed as sebaceoma (Supplementary Figure). In addition, 18 months after the initial operation, periodic CT detected local recurrence at the left abdominal wall with a 5.0-cm soft tissue mass, and second wide resection of the recurrent tumor was performed. The resected tumor showed an identical histopathological phenotype to the primary myxofibrosarcoma. At the final follow-up, one year after the reoperation, there was no evidence of recurrence or metastasis.

**Fig. 2:**
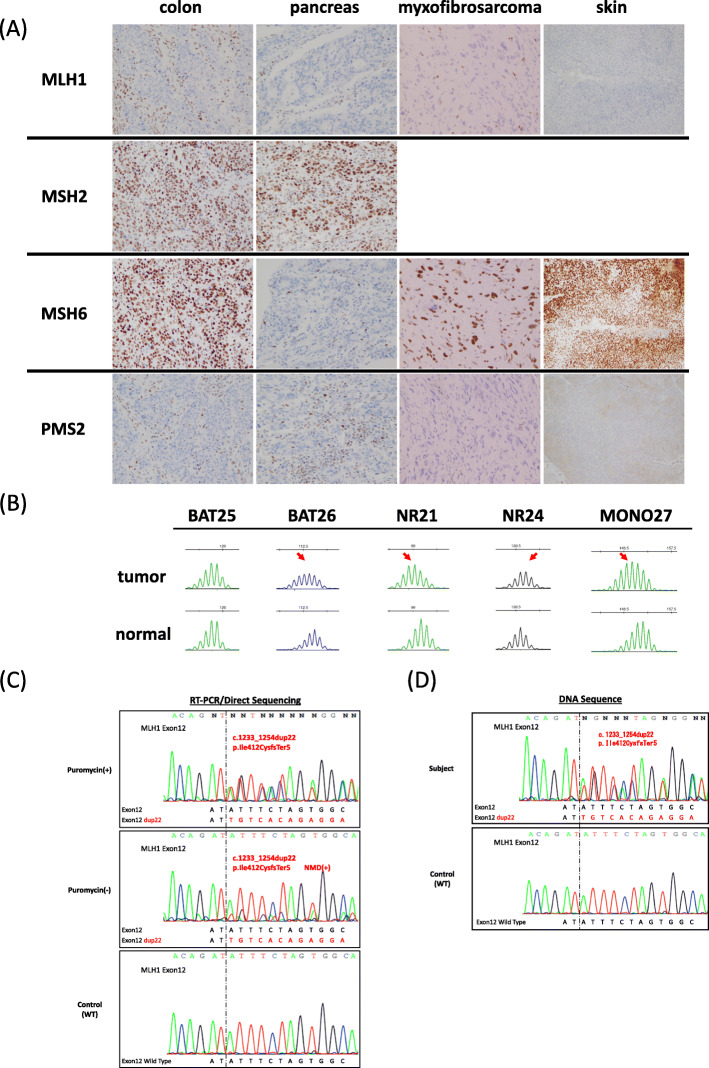
Screening of Lynch syndrome and germline genetic testing for MMR genes of the patient. **A** Immunohistochemistry (IHC) of four tumors using four antibodies to mismatch repair (MMR) proteins. IHC of MSH2 with myxofibrosarcoma and cutaneous tumor was skipped in this screening step. **B** MSI analysis with the Promega panel. Red arrows represent abnormal microsatellite markers. **C** RT-PCR/direct sequencing of the *MLH1* gene. The upper panel indicates the sequencing profile from the puromycin-treated total RNA sample. The middle panel shows the sequencing profile without puromycin treatment. The lower panel shows the sequencing profile of wild-type controls. **D** DNA sequencing analysis of the *MLH1* gene of the patient (top) and the wild-type control (bottom)

This patient (Proband, familial number: III-4) had a history of rectal cancer (at 47 years old), transverse colon cancer (at 51 years old), duodenal cancer (at 54 years old), descending colon cancer (at 71 years old), and pancreatic cancer (at 72 years old) (Fig. 1D). Both descending colon and pancreatic cancer were moderately differentiated adenocarcinoma, and the stages were IIA and III, respectively. His brother (III-2) developed colorectal cancer (at 42 years old), and his sister (III-3) had colorectal cancer (at 42 years old), endometrial cancer (in her 40’s), and pancreatic cancer (at 66 years old), ultimately dying at 68 years old. His maternal uncle (II-4) also had colorectal cancer and died at 60 years old. He met both the Amsterdam II criteria for identifying LS and the revised Bethesda guidelines for testing colorectal tumors for microsatellite instability (MSI) [15, 16]. We therefore performed an IHC analysis of MMR proteins using surgical specimens from his descending colon cancer, pancreatic cancer, myxofibrosarcoma, and sebaceoma (Fig. 2A).

The loss of expression of MLH1 and PMS2 proteins was detected in all tumor cells, whereas the expression of MSH2 and MSH6 was retained, although the expression of MSH6 was attenuated in the pancreatic tumor, suggesting that all of his tumors had an *MLH1* genetic alteration. In addition, an MSI analysis in tissues from myxofibrosarcoma with the Promega panel (BAT25, BAT26, NR21, NR24, MONO27) revealed MSI-H, with four out of five microsatellites being unstable (Fig. 2B). We subsequently confirmed MLH1 promoter unmethylation in tissue samples of myxofibrosarcoma by Na-bisulfite PCR/SSCP.

Furthermore, after obtaining consent, germline genetic testing for the MMR genes was performed. RT-PCR/direct sequencing detected a 22-bp duplication in exon 12 of the *MLH1* gene, resulting in a frameshift variant (NM_000249.4: c.1233_1254dup22, p.Ile412CysfsTer5) (Fig. 2C). Signals from the mutated allele were enhanced in puromycin-treated samples, showing the presence of nonsense-mediated mRNA decay in samples without puromycin treatment. This variant was validated in the analysis using genomic DNA samples (Fig. 2D). Although this variant was not reported in ClinVar, it was regarded as a pathogenic variant because of interrupting protein synthesis (PVS1 in ACMG/AMP guideline) and absence in population database (PM2). In the analysis using MLPA, no difference in the total copy numbers of *MLH1* was observed.

Based on these results, the patient was diagnosed with MTS, a subtype of LS, with the *MLH1* PGV. His son (IV-7) and daughter (IV-8) underwent genetic testing, but the frameshift variant of *MLH1* gene observed in the proband was not detected in either of them (Fig. 1D).

## Discussion and conclusions

We herein report detailed clinical, pathological, and molecular data of a myxofibrosarcoma with a sebaceous tumor that occurred synchronically in a patient with LS, leading to a diagnosis of MTS. Of note, all of the tumor cells, including those from descending colon cancer, pancreatic cancer, myxofibrosarcoma, and sebaceoma, lacked the immunohistochemical expression of both MLH1 and PMS2, indicating all of these tumors are LS-associated tumors.

There have been few reports of LS-related sarcoma, with only 47 sporadic cases reported in the past [11]. The most frequent location of LS-associated sarcoma is in the extremities (18 cases), followed by the trunk (5 cases), uterus (5 cases), and brain (3 cases) [11]. Major histological subtypes were as follows: seven cases of liposarcoma, six of leiomyosarcoma, four of malignant fibrous histiocytoma, and three of rhabdomyosarcoma and osteosarcoma [11]. Only one LS-related myxofibrosarcoma has been reported with an *MLH1* germline variant (*MLH1* c.678-7_686del16) [17]. MTS-related sarcoma is extremely rare, and only five cases have been reported in the literature, including undifferentiated pleomorphic sarcoma, pleomorphic liposarcoma, osteosarcoma, and two sarcomas (not specified) (Table 1) [5, 9–11]. To our knowledge, this is the first case of a high-grade myxofibrosarcoma of the abdominal wall associated with MTS.

**Table 1:** Characteristics of sarcoma patients with MTS

Study	Age (years)	Sex	Tumor site	Tumor histology	Other malignancies	MMR loss (IHC)	MSI	MMR germline variant
Lee et al. [10]	58	M	Retroperitoneum	Undifferentiated pleomorphic sarcoma	Colon, Sebaceous neoplasm	MSH2	MSI-H	*MSH2* (not specified)
Yozu et al. [5]	74	M	Buttock	Pleomorphic liposarcoma	Colon, Sebaceous neoplasm, Prostate	MSH2/MSH6	NA	*MSH2* (not specified)
Latham et al. [9]	71	NA	NA	Soft tissue sarcoma	Colon, Endometrial, Sebaceous adenoma	MSH2/MSH6	MSI-H	*MSH2* (c.1216 C > T, p.Arg406Ter)
de Angelis de Carvalho et al. [11]	40	F	NA	Osteosarcoma	Sebaceoma	MSH2/MSH6	MSI-H	*MSH2* (c.1661 + 1G > A, p.Gly504Alafs*3)
	51	M	NA	Soft tissue sarcoma	Colon, Sebaceoma	MSH2/MSH6	NA	*MSH2* (c.1444 A > T, p.Arg482Ter)
Present case	73	M	Abdominal wall	Myxofibrosarcoma	Colon, Duodenal, Pancreas, sebaceous epithelioma	MLH1/PMS2	MSI-H	*MLH1* (c.1233_1254dup22, p.Ile412CysfsTer5)

*MMR* mismatch repair, *IHC* immunohistochemistry, *MSI* microsatellite instability, *MSI-H* high-frequency microsatellite instability, *NA *not available

The Prospective Lynch Syndrome Database has reported that the probability of subsequent tumors, including sarcoma, following the first tumor increases with increasing age [18, 19]. Therefore, as life expectancy increases, the number of LS-related sarcomas, including myxofibrosarcoma, may increase in the future. Furthermore, the major PGV of LS-related sarcomas are *MSH2* (58.1 %), followed by *MLH1* (27.9 %) [11]. Most families with MTS also have pathological variants in *MSH2* and *MLH1* [3], and about three-fourth patients belonging to a families with the *MSH2* gene c.942 + 3 A > T PGV are diagnosed with MTS [4]. Interestingly, our patient has an *MLH1* PGV, whereas all five previously reported sarcomas with MTS harbored the *MSH2* variant (Table 1) [5, 9–11]. Therefore, we reported the case of MTS-related myxofibrosarcoma in a carrier of the *MLH1* PGV, a finding that differed from the cases described in previous reports.

MSI-H/MMR-deficient solid tumors have recently been suggested to be susceptible to immune checkpoint blockade [20, 21]. Indeed, the efficacy of pembrolizumab, an anti-programmed-death-1 (anti-PD-1) antibody, in 94 MSI-H solid tumors was reported in the phase II KEYNOTE-158 study [22]. The objective response rate was as high as 34.3 % (95 % confidence interval: 28.3–40.8 %). Among these 94 cases, only 1 case of soft tissue sarcoma showed very high efficacy of pembrolizumab. Another case reported a metastatic chemo-resistant pleomorphic rhabdomyosarcoma associated with LS that showed a complete response after the administration of the anti-PD-1 antibody nivolumab [23]. However, there are likely very few sarcoma patients who are eligible for anti-PD1 antibody; for example, there were only 7 MSI-H sarcoma patients among 3256 (0.21 %) in a previous study [24]. Another study reported that only 2.3 % (7/304) of sarcomas showed MMR deficiency [25]. In addition, the effectiveness of anti-PD-1 antibodies against myxofibrosarcoma remains unknown, although some studies have described the efficacy of pembrolizumab for such rare tumors [26–28]. The present case developed local recurrence 18 months after the primary surgery. As there is a risk of further local recurrence or metastasis in the future, we should continue to watch the patient carefully with periodic examinations. When the tumor progresses again, we can consider the use of immune checkpoint inhibitors for this rare phenotype of MSI-H myxofibrosarcoma, even in this elderly patient [22].

In summary, we documented the first case of MSI-H myxofibrosarcoma with MTS in an *MLH1* PGV carrier. Although rare, we should recognize that sarcomas can be part of the spectrum of LS and MTS. These lesions may be highly susceptible to immune checkpoint inhibitors, despite usually showing chemoresistance against systemic therapy.

## Supplementary Information

**Additional file 1.**

## Data Availability

Not applicable.
