# Supplementary material for: Myxofibrosarcoma harboring an MLH1 pathogenic germline variant associated with Muir-Torre syndrome: a case report
Source: Hered Cancer Clin Pract. 2021 Aug 21;19:34. doi: 10.1186/s13053-021-00192-z (PMC8379813; doi:10.1186/s13053-021-00192-z)
Supplement: Supplementary file 1 — Additional file 1. [file 13053_2021_192_MOESM1_ESM.pdf]

Supplementary Figure

(A)

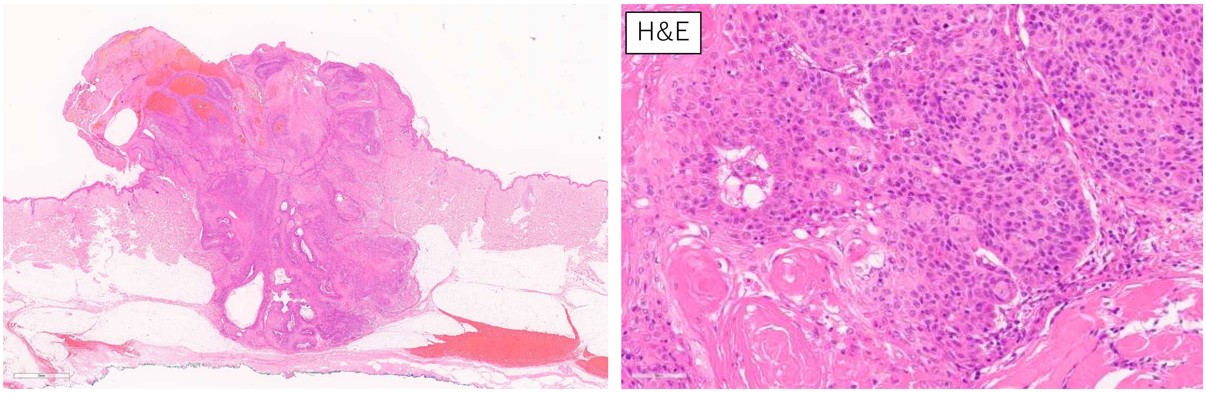

(B)

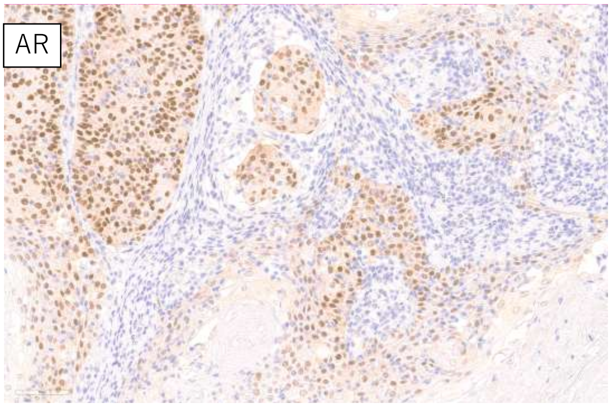

(C)

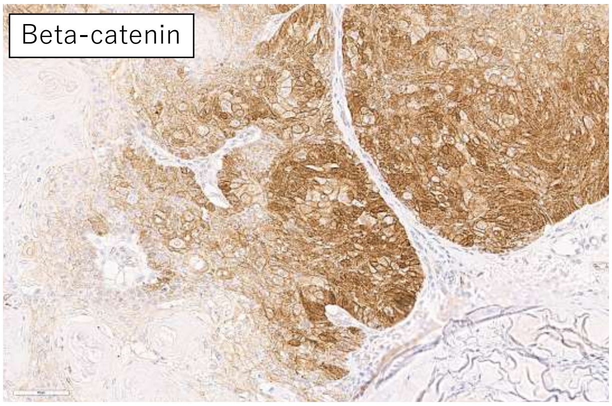

Supplementary Figure. Representative histology of the cutaneous tumor.
(A) Hematoxylin eosin staining of the cutaneous tumor. (B) IHC of androgen receptor (AR). AR is diffuse positive. (C) IHC of β-catenin. β-catenin is focally positive in the nuclei.
